# PARP3 promotes macrophage inflammation via mono ADP ribosylation of Ppia Glu140

**DOI:** 10.1186/s10020-025-01278-3

**Published:** 2025-06-03

**Authors:** Runjie Fan, Rongxing Zhu, Xiangxiu Cao, Shuhui Ye, Fengyi Gao, Yue Wu, Wanxin Yao, Guang Liang, Yanmei Zhang

**Affiliations:** 1 School of Laboratory Medicine and Bioengineering, Hangzhou Medical College, Hangzhou, 310012 P.R. China; 2 School of Pharmacy, Hangzhou Medical College, Hangzhou, 310012 P.R. China; 3 Key Laboratory of Biomarkers and In Vitro Diagnosis Translation of Zhejiang Province, Hangzhou, 310063 Zhejiang China

**Keywords:** PAPR3, Ppia, NF-κB, ALI, Mono-ADP ribosylation

## Abstract

**Background:**

Acute lung injury (ALI) carries significant mortality with limited targeted therapies. Macrophages drive early inflammatory propagation in ALI, exacerbating pulmonary inflammation. While ADP-ribosylation is a dynamic and reversible post-translational modification (PTM) associated with inflammatory diseases, its role in macrophage-mediated inflammation remains unclear.

**Methods:**

Murine ALI model was established via intratracheal instillation with lipopolysaccharide (LPS). The ALI lung tissues and cultured mouse macrophage line (RAW264.7) treated with LPS were used to assess the expression of poly ADP-ribose polymerases (Parps). RNA sequencing (RNA-seq) identified differentially expressed genes (DEGs) following *Parp3* knockdown (siParp3) in LPS-stimulated RAW264.7 cells, with subsequent pathway analysis was via transcription factors (TFs) profiling and gene ontology (GO) enrichment. In RAW264.7 cells, *Parp3* and peptidyl-prolyl cis–trans isomerase A (*Ppia*) was modulated by siRNA or plasmid transfection. PARP3-Ppia interaction and ADP-ribosylation were assessed by immunoprecipitation. Modification alterations due to mutations at Ppia modification sites were assessed by immunoprecipitation. Enzyme-linked immune sorbent assay (ELISA) was used to quantify Ppia secretion. A mouse ALI model was used to evaluate the lung-protective and therapeutic effects of PARP3 inhibitor ME0328 by detecting inflammatory cytokines, phosphorylation of p65 and lung histopathology.

**Results:**

LPS induced the expression of Parp3 in RAW264.7 cells and ALI lung tissues, correlating with elevated inflammatory cytokines. The 52 overlapping DEGs were mainly enriched in Toll-like receptor (TLR) signaling pathway. PARP3 promoted inflammation via NF-κB activation. ME0328 blocked NF-κB pathway activation in RAW264.7 cells and lung tissues. Immunoprecipitation confirmed that PARP3 interacted with Ppia. Ppia was modified with mono ADP-ribosylation. Ppia-E140 was the most inflammation related modification site. The mutation of E140 inhibited inflammatory response, mono ADP-ribosylation and secretion of Ppia. In vivo, ME0328 reduced inflammatory response, alleviated pulmonary edema and mitigated histopathological damage.

**Conclusions:**

We identified the NF-κB as the downstream signaling pathway mediated by Ppia for PARP3 to promote macrophage inflammation. ME0328 alleviated pulmonary inflammation through the NF-κB signaling pathway. Our findings provide evidence that macrophage inflammation is associated with the mono ADP-ribosylation on Ppia. Understanding mono ADP-ribosylation regulation in macrophage from ALI may provide insight into the pro-inflammatory mechanisms and opportunities for effective therapeutic to treat acute lung injury.

## Introduction

Inflammation is a basic immune response triggered by infection or damage in tissues (Greten et al. 2019; Huang et al. 2020). Acute lung injury (ALI) can be caused by indirect reasons (chest trauma, pancreatitis, blood transfusion and burns), or direct pulmonary infection and trauma. Diffuse pulmonary infiltration and alveolar injury, massive infiltration of neutrophils and alveolar macrophages, edema and necrosis of lung tissue are the main pathological features about ALI (Bos et al. 2022; Matthay et al. 2005). There are no effective targeted strategies for ALI, only ordinary supportive treatment and antibiotic administration are used for improving the condition (Bos et al. 2022). Targeted therapy for inhibiting the inflammatory pathway and suppressing the inflammatory storm is an effective method to relieve ALI (Butt et al. 2016). Alveolar macrophages play a role in the pathogenesis of ALI. As the first line of defense against injury in the lungs, alveolar macrophages are regarded as one of the most important cell types in the lungs’ response to injury and are involved in the occurrence and prognosis of ALI (Yao 2003). Macrophage inflammatory protein-2 (MIP-2) produced by alveolar macrophages is an effective chemoattractant for neutrophils, inducing the migration of neutrophils to the inflammatory site (Gupta et al. 1996). This process emphasizes that macrophages play a major role in acute lung injury, while neutrophils are effector cells. Macrophages actively release cytokines and reactive oxygen species (ROS), aggravating endothelial cell damage and leading to increased vascular permeability and pulmonary edema (Osorio-Valencia and Zhou 2024). The interaction between macrophages and endothelial cells is crucial in the progression of ALI, but macrophages often serve as a drive force. Fibroblasts support inflammation but are secondary to macrophages. Although fibroblasts are involved in the inflammatory process by secreting chemokines such as C-X-C motif chemokine ligand 10 (CXCL10) and help recruit macrophages, they mainly affect the later stages of injury and repair (Tsai et al. 2019). Epithelial cells also participate in the inflammatory process by releasing micro-vesicles (MVs), but these micro-vesicles are mainly used to activate macrophages and further spread inflammation. However, once activated, macrophages are the main mediators of the pro-inflammatory response (Lee et al. 2016). This highlights that macrophages, rather than epithelial cells, are instrumental in the inflammatory drive during ALI. Macrophages can be divided into two distinct polarization states: classically activated phenotype (M1), and the alternatively activated phenotype (M2), the former is closely linked to pro-inflammatory responses, while the latter plays a key role in anti-inflammatory reactions (Sica et al. 2012). Macrophage M1 phenotype is involved in the acute phase (Johnston et al. 2012). Stimulated by factors such as microRNA, M1 pro-inflammatory macrophages are activated (Ying et al. 2015), generating a large amount of pro-inflammatory cytokines to promote LPS-induced ALI (Xiao et al. 2015). The differences in the timing and degree of polarization among macrophages influence the prognosis and severity of ALI (Gill et al. 2017). The expression or posttranslational modifications (PTMs) of important factors in macrophages that promote inflammation could be potential targets for ALI therapy.

PTMs regulate a variety of biological and pathological processes, including DNA repair, cell differentiation, gene transcription, signal transduction pathways, energy metabolism, and epigenetics (Chambon et al. 1963, Bai. 2015, Pazzaglia and Pioli 2019). PTMs like phosphorylation, ubiquitination, acetylation has been studied a lot (Hong et al. 2018). PTMs play an important regulatory role in ALI. The post-translational modifications of important members in many inflammatory pathways have a major part in activating the inflammatory pathway. The phosphorylation of STAT3 continuously activates M1 pro-inflammatory macrophages, leading to the massive release of pro-inflammatory cytokines and inhibiting the anti-inflammatory response (Zou et al. 2020; Johnson et al. 2018). The phosphorylation of ERK causes vascular endothelial injury (Chen et al. 2017), and the phosphorylation of Krt7 (Keratin 7) S53 leads to barrier dysfunction of alveolar epithelial cells (He et al. 2023). N-acetyltransferase (NAT) 10-mediated TFRC-Ac4c acetylation aggravates ALI by promoting ferroptosis (Xing et al. 2024). Methylation of DNA and miR-1246 promoter exacerbates inflammatory responses via targeting KLF7 (Krueppel-like factor 7) and underlies monocyte dysregulation and immune exhaustion memory in ALI (Xu et al. 2021; Caldwell et al. 2024). Compared with other PTMs, ADP-ribosylation, especially mono-ADP-ribosylation, has received relatively little attention. PARPs (also known as ADP-ribotransferase diphtheria toxin-like ARTDs) catalyze ADP-ribosylation (Ke et al. 2019; Vitali et al. 2023). The PARP family of factors includes 17 PARPs (Karicheva et al. 2016; Bieche et al. 2013, Schreiber, et al. 2006) and is classified into poly-ADP‒ribose polymerase (PARP) and mono-ADP‒ribose transferase (MART) (Vyas et al. 2014; Yang et al. 2022; Quan et al. 2015). PARP1, PARP2, PARP5a and PARP5b generate poly-(ADP-ribose), and the majority of other PARPs generate mono-ADP-ribose (Bieche et al. 2013; Schreiber et al. 2006; Sun et al. 2014). The WGR (recognition domain) and CAT (catalytic domain) of PARPs transfer ADP-ribose from NAD^+^ to amino acid residues of target proteins to form mono- (MARylation) or poly-ADP-ribosylation (PARylation). PARP1 as a therapeutic cancer target, has been reported to play a role in inflammatory diseases (Wang et al. 2021; Wennerberg et al. 2022). However, mono-ADP-ribosylation, has not been well studied in these diseases (Di Girolamo et al. 2018; Feijs et al. 2013; Fabrizio et al. 2015).

As a mono-ADP-ribosyl transferase (Grundy et al. 2016; Langelier et al. 2014; Loseva et al. 2010), PARP3 is usually activated in the presence of notched DNA and catalyzes mono-ADP-ribosylation on glutamate residues (Glu or E) of substrate proteins such as histones H1 and H2B (Ewing et al. 2007). PARP3 is not only involved in the repair of DNA damage in cancer cells but also regulates cell mitosis (Boehler et al. 2011). ME0328 is the selective inhibitor of PARP3. Although, PARP3 has been reported to be associated with cancer, research data on the crosstalk between PARP3 and inflammatory diseases is lacking to date. Cyclophilin A (Ppia, CypA) was a widely established proinflammatory agent (Sun et al. 2014; Luan et al. 2021; Liu et al. 2023) that enhances the stability of the NF-κB p65 subunit and promotes its nuclear translocation (Sun et al. 2014; Dongsheng et al. 2017) to increase the production of proinflammatory cytokines. Ppia is involved the process of antiviral immunity (Liu et al. 2017), and positively regulates IL-6 trans-signaling (Luan et al. 2021).

In this research, we revealed that Ppia is identified as a substrate of PARP3 and Ppia mono-ADP-ribosylation by PARP3 enhancing the transcription activity of NF-κB and promoting ALI development. Elimination of the mono-ADP-ribosylation site of Ppia impaired its modification and proinflammatory function by PARP3. It is demonstrating a correlation between MARylation and inflammation. These findings suggested that targeting a PARP3/Ppia/NF-κB signaling circuit with PARP3 inhibitor, alone or with NF-κB antagonists, might be a potential therapeutic for PARP3-overexpressing in ALI.

## Materials and methods

### Reagents, antibodies and plasmids

Lipopolysaccharide (Cat. #L4391) was purchased from Sigma, ME0328 was purchased from Selleck (Cat. #S7438), anti-Parp3 (Cat. #11289-1-AP), anti-Ppia (Cat. #10720-1-AP), anti-HA (Cat. #51064-2-AP), anti-Flag (Cat. #20543-1-AP), anti-Gapdh (Cat. #60004-1-Ig) were purchased from Proteintech, anti-p-P65 (Cat. #3033S), anti-P65(Cat. #8242S) were purchased from CST, anti-IgG (Mouse, Cat. #A7016), anti-IgG (Rabbit, Cat. #A7028) were purchased from Beyotime, anti-mono-ADPr Binding Reagent was purchased from Millipore (Cat. #MABE1076), β-nicotinamide adenine dinucleotide (NAD^+^, Cat. #HY-B0445) was purchased from MCE, Escoli receptor DH5α&BL21(DE3) was purchased from Accurate Biology, and His label protein purification kit (Reduction resistant Chelate type) 10 mL (Cat. #P2226) and Bradford protein concentration assay kit (Cat. #P0006) were purchased from Beyotime, Endotoxin-free plasmid extraction kit (Cat. #BW-PD1222-02) was purchased from BiomegaPurified proteins Ppia-WT and Ppia-E140A were purchased from Novan Biology, overexpressed plasmids PARP3-HA, Ppia-Flag were purchased from Youbio Biology, and siRNA of Parp3, Ppia was purchased from GenePharma (https://www.genepharma.com/). Liposome transfection reagent Lip2000 was purchased from Biosharp, siRNA transfection reagent CALNP™ RNAi in vitro was purchased from Beijing Dona Pharmaceutical. Protein Marker was purchased from Vazyme (Cat. #MP102), Mouse CypA ELISA Kit was purchased from CUSABIO (Cat. #CSB-E09282 m).

### Cell culture

RAW 264.7 and NIH-3T3 cells were purchased from ATCC. The cells were cultured in a humidified CO_2_ (5%) incubator at 37 ℃ RAW264.7 and NIH-3 T3 cells were cultured in DMEM high glucose (Bio-channel) supplemented with 10% FBS (Bio-channel). The cells were cultured in a 10% FBS complete DMEM for 1 to 2 d, then reseeded into a 6 or 12 well plate overnight in a complete DMEM at a rate of 10^6^ or 5 × 10^5^ cells per well, followed by LPS stimulation, drug treatment, and transfection the next day.

### Cell transfection

The cells were inoculated into 6-well plates (1 × 10^6^•mL^−1^) and 12-well plates (5 × 10^5^•mL^−1^) and cultured in a humidified CO_2_ (5%) incubator at 37 ℃ until they reached 70% confluent. Lipofectamine 2000 transfection reagent (Biosharp) and siRNA transfection reagent CALNP™ RNAi are used for plasmids and siRNA, respectively. The cells were harvested and analyzed 48 h after transfection. The transfection efficiency was determined by RT-qPCR analysis. The target sequences of the two siRNAs were listed as follows: si-Parp3: sense-5’-GGCAAAGAGCACCACAUCATT-3’, anti-sense-5’-UGAUGUGGUGCUCUUUGCCTT-3’; si-Ppia: sense-5’-GGUGACUUUACACGCCAUATT-3’, anti-sense-5’-UAUGGCGUGUAAAGUCACCTT-3’.

### Animal model of ALI

Two months-old adult male C57BL/6 (20 g) from Animal Center at Hangzhou Medical College. All mice were placed in a temperature and light-controlled room of 25 ℃ and fed standard laboratory food. The mice were randomly divided into 3 groups: phosphate buffered saline (PBS) group, LPS group, LPS + ME0328 group (*n* = 5 per group). Mice were anesthetized with 0.3% sodium pentobarbital, and 5 mg•kg^−1^ LPS (purchased from Sigma, dissolved in saline) was intratracheal for 6 h to induce ALI models. 1 h before LPS treatment, 10 mg•kg^−1^ ME0328 was intraperitoneally injected. Eye blood, lung tissue, and bronchoalveolar lavage fluid (BALF) were harvested and retained for subsequent testing.

### Immunofluorescence (IF)

The lungs from mice were fixed at 4% PFA (4% paraformaldehyde) at 4 ℃ for 24 h and embedded with paraffin. Sections of lung tissue were performed using a paraffin section machine (Thermo, HM325). The tissue was washed three times with 1 × PBS to remove paraffin, and non-specific binding was blocked with 10% BSA (with 1 × PBS) at 37 ℃ for 30 min. Then, the tissue sections were added with primary antibody (CD68, CST, 97778, 1:500; Parp3, Proteintech, 11289-1-AP, 1:50) and incubated at 4 ℃ overnight. On the second day, sections were rinsed 3 times with 1 × PBS, corresponding secondary antibody (Alexa Fluor 488 or 594) was added, and incubated at 37 ℃ for 45 min. Then it was washed three times with 1 × PBS and fixed with anti-fluorescence quencher containing DAPI. Olympus BX53 biological microscope was used to collect the images of each section.

### Test for lung wet/Dry (W/D) weight ratio

To assess the degree of edema in the lung tissue, the upper right lobe of the lung tissue was separated immediately after molding and the surface fluid and blood were absorbed by filter paper and placed in a pre-weighed tinfoil cap. The tissue sample was placed in an oven at 60 ℃ for 48 h to obtain dry weight, and the W/D ratio was then calculated using the following calculation method: Lung W/D weight ratio = wet weight/dry weight.

### Histology

The lung tissue was fixed overnight with 4% paraformaldehyde at room temperature and handed over to Biossci (Hubei) Biotechnologies Company for Paraffin-embedded lung sections were stained with H&E or Masson and then white-light scanning was performed.

### Enzyme-linked immunosorbent assay (ELISA) determination of IL-6&TNF-α

According to the manual, the TNF-α & IL-6 Mouse Uncoated ELISA kit is first coated with the captured antibody on the enzymic labeled plate overnight at 4 ℃, and the mouse serum and the supernatant of BALF after centrifuging are also stored at 4 ℃. On the second day, washing, sealing, sample loading, incubation, antibody detection, HRP enzyme coupling and TMB color development, and dilute sulfuric acid termination were performed. The absorbance values for each well were obtained at a wavelength of 450 nm by the enzyme-labeler, and then subsequent analysis was carried out.

### The detection method of Ppia secretion in cell culture medium

According to the manual (CUSABIO, Cat. #CSB-E09282m), the 100 μL of standard and samples were added per well. Then the liquid of each well was removed after incubating for 2 h at 37 ℃. The 100 μL of Biotin-primary antibody (1 ×) was added to each well and incubated for 1 h at 37 ℃. Washing and repeating the process two times for a total of three washes. Next, 100 μL of HRP-avidin (1 ×) was added to each well and incubate for 1 h at 37 ℃. Last, 90 μL of TMB was added and incubated for 15-30 min at 37 ℃. And 50 μL of Stop Solution was used for termination. The absorbance values of each well were obtained at a wavelength of 450 nm by the enzyme-labeler, and then subsequent analysis was carried out.

### Quantitative PCR (QPCR)

Total RNA was extracted from cell and lung tissue homogenates using the SteadyPure RNA Extraction Kit (Accurate Biology) according to the manufacturer's instructions. According to the manufacturer's instructions, cDNA was synthesized using the Evo M-MLV reverse transcription Premix kit (Accurate Biology) from 1 μg total RNA. Oligonucleotide (dT) primers and SYBR Green Pro Taq HS premixed qPCR kit (Accurate Biology) were used to analyze relative gene expression by qPCR. qPCR primer pairs are listed below. The Ct values generated by Bio-Rad CFX Manager 3.1 were analyzed using the 2^−△△Ct^ method. The expression values of target genes were normalized with reference to the expression values of GAPDH. The primer sequences are listed as follows: Gapdh, 5’-CATCACTGCCACCCAGAAGACTG-3’ and 5’-ATGCCAGTGAGCTTCCCGTTCAG-3’; PARP3-homo, 5’-TCTCTGAGCAGGAGAAGACGGT-3’ and 5’-TGTGGTTGCTGCCAGTCTGTTC-3’; PARP3-Mus, 5’-GGAGAAAGTGGAGAGAGGTGCCA-3’ and 5’-GCCAGTCTGTTTCAGGTAGGTC-3’; Ppia, 5’-CATACAGGTCCTGGCATCTTGTC-3’ and 5’-AGACCACATGCTTGCCATCCAG-3’; TNF-α, 5’-TGATCCGCGACGTGGAA-3’ and 5’-ACCGCCTGGAGTTCTGGAA-3’; IL-1β, 5’-TTCAGGCAGGCAGTATCACTC-3’ and 5’-GAAGGTCCACGGGAAAGACAC-3’; IL-6, 5’-GAGGATACCACTCCCAACAGACC-3’ and 5’-AAGTGCATCATCGTTGTTCATACA-3’.

### Co-immunoprecipitation and immunoblot analysis

The cells were harvested and lysed under ice in a Radio-Immunoprecipitation Assay (RIPA) buffer (Beyotime) containing a protease inhibitor cocktail (Beyotime) lysis buffer. After adding the above-mentioned lysate and steel ball to the lung tissue, it was homogenized in a high-throughput tissue grinder. Then the cell and tissue sample were centrifuged at 4 ℃, 13000 g•min^−1^ for 20 min. The Bradford protein concentration assay kit (Beyotime) was used to quantify proteins. And then equal amounts were separated on sodium dodecyl sulfate–polyacrylamide (SDS–PAGE) gel and transferred to the 0.22 μm PVDF membrane (Millipore). The membranes were blocked with protein-free rapid blocking solution at room temperature for 20 min and incubated with primary antibodies with a gentle shake at 4 °C overnight. The PVDF membrane was then incubated with indicated secondary antibody at room temperature for 1 h. The protein bands on membranes were then analyzed using the hypersensitive ECL chemiluminescence kit (NCM Biotech) and developer (Bio-Rad). The bands were scanned with Image J software to calculate grayscale. For co-immunoprecipitation, the 500 μg of precleared cell lysates lysed by IP buffer (50 mM Hepes, 150 mM NaCl, 10% Glycerol, 0.1% Triton X-100, 20 mM MgCl_2_, 1% EDTA) at least was incubated with IP antibody at 4 ℃ overnight, and the next day was incubated with Protein A+G magnetic beads (Beyotime) at 4 ℃ for 3 to 4 h. After incubation, the magnetic beads were washed three times with 1 × TBS (including PMSF), and the magnetic beads were eluted with 1 × TBS diluted protein loading buffer. Eluent and whole cell extract were separated on SDS-PAGE gel and then bands were detected by western blotting with corresponding antibodies.

### Transcriptome sequencing analysis

RAW264.7 cells were stratified into three plates (5 × 10^5^•mL^−1^), transfected with siRNA for 48 h, and stimulated with LPS for 6 h. According to the manufacturer's instructions, total cell RNA was prepared using the SteadyPure RNA extraction kit (Accurate Biology), and then reverse transcription and qPCR were performed to verify the treatment effect. Briefly, 10 μg total RNA was used to purify poly(A) RNA. Samples were fragmented and reverse transcribed to create cDNA libraries. cDNAs were purified following uracil-DNA glycosylase (UDG) enzyme digestion and PCR, and the final paired-end libraries were formed (PE150). Paired-end sequencing was performed using DNBSEQ. With the final transcriptome generated, Bowtie2 and RSEM were executed to estimate the expression levels for mRNAs by calculating fragments per kilobase of exon per million mapped fragments (FPKM). A reference transcriptome analysis was performed using the MGI-SEQ platform (DNBSEQ-50 platform, BGI-Shenzhen, China). Differential gene screening and pathway analysis were performed using the Dr. Tom online system (http://biosys.bgi.com). These sequence data have been submitted to the NCBI databases under accession no. PRJNA1224952 (https://www.ncbi.nlm.nih.gov/bioproject/?term=PRJNA1224952).

### Isolation of Mouse Primary Peritoneal Macrophages (MPMs)

The mice were sacrificed by cervical dislocation and soaked in 75% alcohol and placed in a biosafety cabinet for subsequent operations. On the right side, a 5 mL syringe was used to slowly inject 4 to 5 mL of serum-free DMEM into the abdomen. After thorough massage, a pipette was used to transfer the fluid from the abdominal cavity and the abdominal cavity was washed repeatedly with serum-free DMEM medium to collect more peritoneal macrophages. After centrifuging, resuspending and cell counting, the cells were reseeded in T25 flask containing DMEM complete medium (containing 10% FBS, 50 U/mL penicillin, and 50 mg/mL streptomycin). After 2 h of the cells were placed in a 37 ℃ cell incubator with 5% CO_2_, the mouse primary peritoneal macrophages (MPMs) were completely adherent, and the medium was then replaced with DMEM complete medium for overnight culture.

### Expression and purification of hPARP3

Using human PARP3 (hPARP3) (NCBI reference sequence: NM_001003931.3) purchased from Youbio Biology as the original plasmid template, primers were designed to amplify the target gene PARP3 and clone it into the bacterial vector pET-28a. The recombinant plasmid pET28a-hPARP3 was transformed into Escherichia coli receptor BL21 (DE3). His labeled hPARP3 was expressed after overnight induction of IPTG with a final concentration of 1 mM at 18 ℃. Next, the bacteria were collected and re-suspended with non-denatured lysate from the protein purification kit, and added with appropriate protease inhibitors, benzoyl fluoride (PMSF) and lysozyme, and ultrasound treated on ice for 20 min. After centrifugation at 15,000 g at 4 ℃ for 20 min, the supernatant was collected and added to a protein Purification column with His tag Purification Resin pre-balanced with non-denaturing lysate. The recombinant hPARP3 protein was eluted twice with 500 μL non-denaturing eluent containing 50 mM imidazole. Protein concentrations were estimated using Coomassie brilliant blue. Finally, the protein after two elution was evenly divided at 100 μL and stored at −80 ℃ for later use. Detection and confirmation of recombinant hPARP3 protein by Coomassie staining and PAGE.

### Mono-ADP ribosylation of Ppia assay

References to concentrations of NAD^+^, dsDNA, and hPARP3 (Ji et al. 2018). NAD^+^, dsDNA, hPARP3, PpiaWT, Ppia E140A and buffer solution (50 mM Tris-HCl, 2 mM MgCl_2_, pH 8.0) were added to the ice bath and incubated at 25 ℃ in a gradient PCR apparatus for 1 h. Appropriate amount of protein loading buffer was added, boiled at 100 ℃ for 5 min, electrophoresis was performed in SDS-PAGE glue, and subsequently anti-mono-ADPr antibody was incubated to detect the modification degree.

### Statistical analysis

Statistical analysis was performed using GraphPad Prism 9 and Microsoft Excel. Data are expressed as the mean ± standard deviation of at least three independent experiments. The unpaired T-test was used to compare the two groups, and the one-way analysis of variance (ANOVA) was used to compare pairwise between the multiple groups. *p* < 0.05 is considered significant.

## Results

### Macrophages express elevated Parp3 under inflammation

**Fig. 1:**
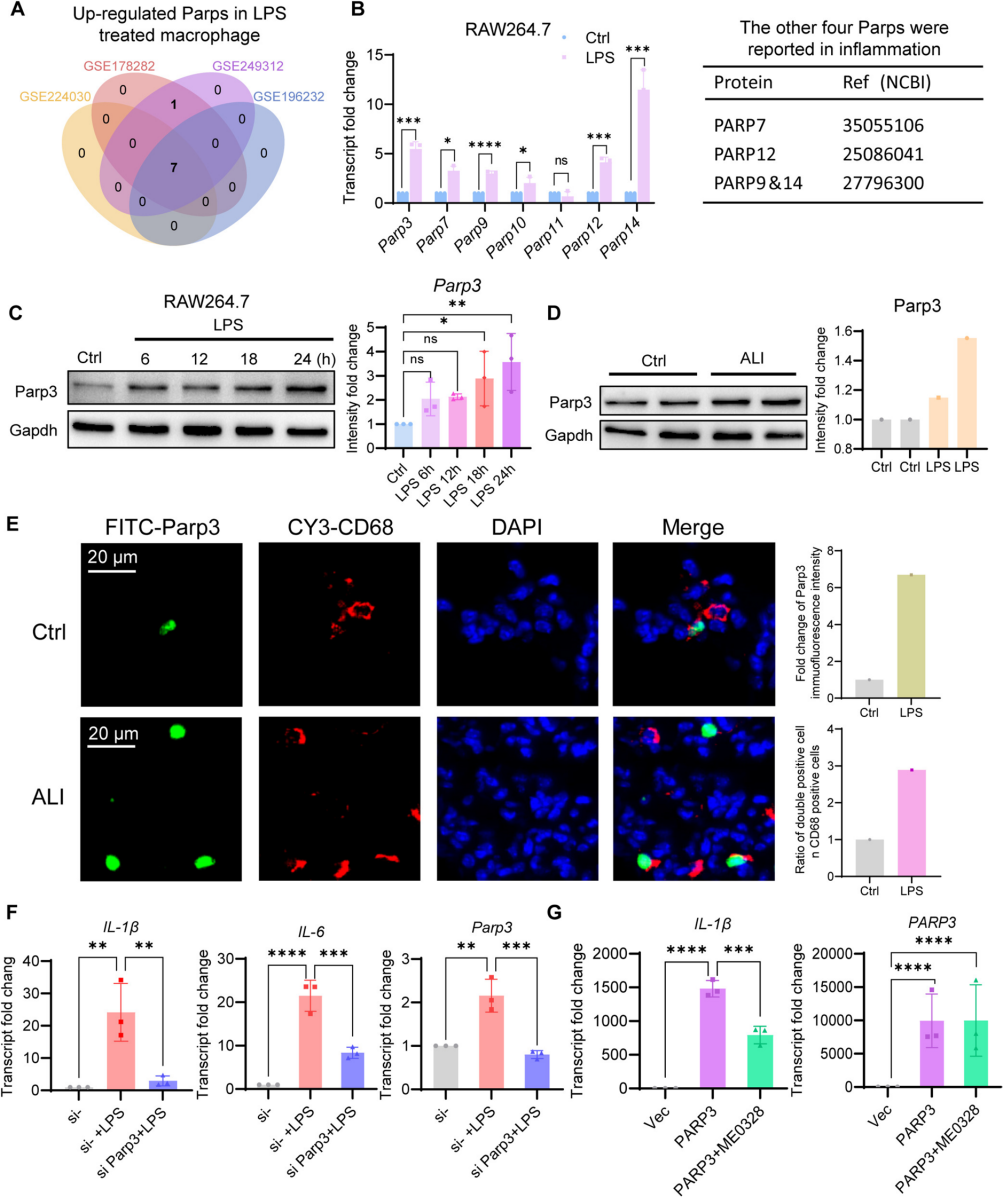
Parp3 increased in response to inflammatory stimulation. **A** The intersection of the four GEO datasets revealed elevated Parps in the inflammatory state. **B** Five members of Parps were induced in RAW264.7 cell by treatment with 6 h of LPS (*n* = 3). **C** Parp3 upregulation was observed in RAW264.7 cell subjected to LPS treatment over time by western blot (*n* = 3). **D** Parp3 expression in ALI lung tissues was detected by western blot and Parp3 expression was higher in mice with ALI lung tissues than in the negative control mice. **E** Representative images of the Parp3 expression in ALI lung tissues detected by immunofluorescence. CY3 as a marker for CD68 was used to detect macrophages. FITC-labeled Parp3 was used to detect the expression and localization of Parp3. **F** *Parp3* knockdown by siRNA or (**G**) Ectopic PARP3 overexpression by plasmid transfection was manipulated in RAW264.7 cells to alter the level of *Parp3*. The expression of *IL-1β*, *IL-6* and *Parp3* was detected by qPCR (*n* = 3). ME0328 was used to inhibit the catalytic activity of PARP3. The data were expressed as the means ± standard deviations. Unpaired T-test was used for statistical analysis in (**B**) and one-way ANOVA was used for statistical analysis in (**C**, **F**, **G**). *p < 0.05, **p < 0.01, ***p < 0.001 or ****p < 0.0001 were considered significant

By consulting public GEO datasets, the common upregulated Parps in the four datasets that described LPS-treated macrophages were summarized. Parp members 3, 7, 9, 12, and 14 were significantly upregulated (Fig. 1A). Few reports on inflammatory diseases exist for Parp7, 9, and 12,14, yet no studies on Parp3 exist. Therefore, we focused on Parp3 in this study. The upregulation of Parp3 expression was verified by quantitative real-time PCR and Western blotting in LPS-treated RAW264.7 macrophages or in lung tissues using the ALI model mice (Fig. 1B-D). Double-label immunofluorescence revealed enrichment of Parp3 within the nucleus of CD68^+^ macrophages in ALI lung tissue. Together with elevated expression of Parp3, the number of double positive cells was increased in ALI lung tissue (Fig. 1E). To understand the importance of Parp3 in macrophage inflammation, *Parp3* expression was knocked down in RAW264.7 cells subjected to LPS treatment. Knockdown of *Parp3* effectively antagonized the proinflammatory effects of LPS (Fig. 1F). *PARP3* overexpression promoted inflammation, and the PARP3 inhibitor ME0328 attenuated this effect (Fig. 1G). The expressions of the classic inflammatory cytokines *IL-1β* and *IL-6* were used to indicate the extent of inflammation.

### Parp3 activated macrophage inflammation via the NF-κB signaling pathway

**Fig. 2:**
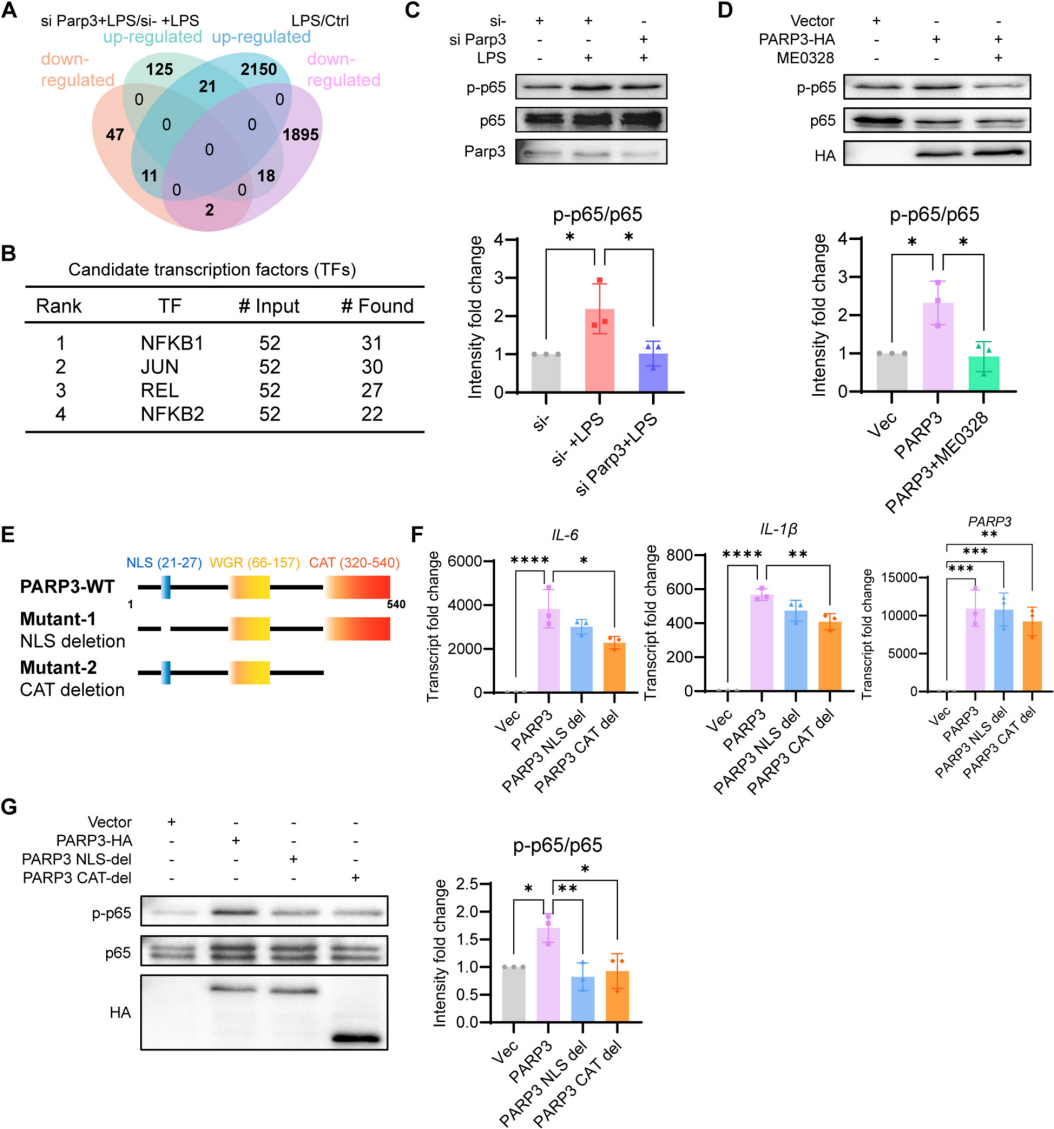
PARP3 activated the NF-κB signaling pathway in macrophages. **A** Venn diagram showed the 52 intersection genes of LPS/Ctrl and siParp3 + LPS/LPS-regulated genes identified via RNA-seq were presented. **B** The transcription factors predicted by the regulated genes from Fig A were listed by rank. **C** *Parp3* knockdown and **D** PARP3 overexpression were performed in RAW264.7 cells to examine the phosphorylation of the p65 subunit of NF-κB by western blot and grayscale analysis was conducted by Image J (*n* = 3). **E** Schematic diagram of NLS and CAT domain deletion mutations in PARP3 overexpression plasmid. **F** The expressions of *IL-1β* and *IL-6* were examined with transfection of the PARP3 mutant plasmids by qPCR (*n* = 3). **G** The phosphorylation levels of p65 were examined following the transfection of wild-type PARP3 and the mutants by western blot (*n* = 3). The results of grayscale analysis of the western blot bands on the left were compared and were presented. The data were expressed as the means ± standard deviations. One-way ANOVA was used for statistical analysis in (**C**, **D**, **F**, **G**). *p < 0.05, **p < 0.01, ***p < 0.001 or ****p < 0.0001 were considered significant

To study the pathways regulated by Parp3, the genes regulated by siParp3 and LPS were analyzed via transcriptome sequencing of RAW264.7 cells. We identified 52 overlapping genes between the two groups (Fig. 2A). The two groups were divided into four Venn diagrams on the basis of upregulation or downregulation. The 52 genes were subsequently used to predict the candidate transcription factors via hTFtarget (https://guolab.wchscu.cn/hTFtarget//). The top four transcription factors involved in the Toll-like receptor signaling pathway were presented in Fig. 2B. The Rel-like domain-containing proteins NFKB1, JUN, REL and NFKB2, which form the NF-κB complex, were displayed in the prediction (Fig. 2B). *Parp3* knockdown suppressed the phosphorylation of the NF-κB p65 subunit (Fig. 2C), while PARP3 overexpression significantly promoted it (Fig. 2D). ME0328 inhibited p65 phosphorylation caused by PARP3 transfection (Fig. 2D).

To further understand the dependence of PARP3 on subcellular localization and catalytic activity, we constructed PARP3 mutants with NLS (nuclear localization sequence) or CAT (catalytic active domain) deletion (Fig. 2E). Both mutations lost the ability to promote inflammatory cytokine expression and the phosphorylation of the NF-κB p65 subunit (Fig. 2F, G).

### Ppia was a substrate of PARP3

**Fig. 3:**
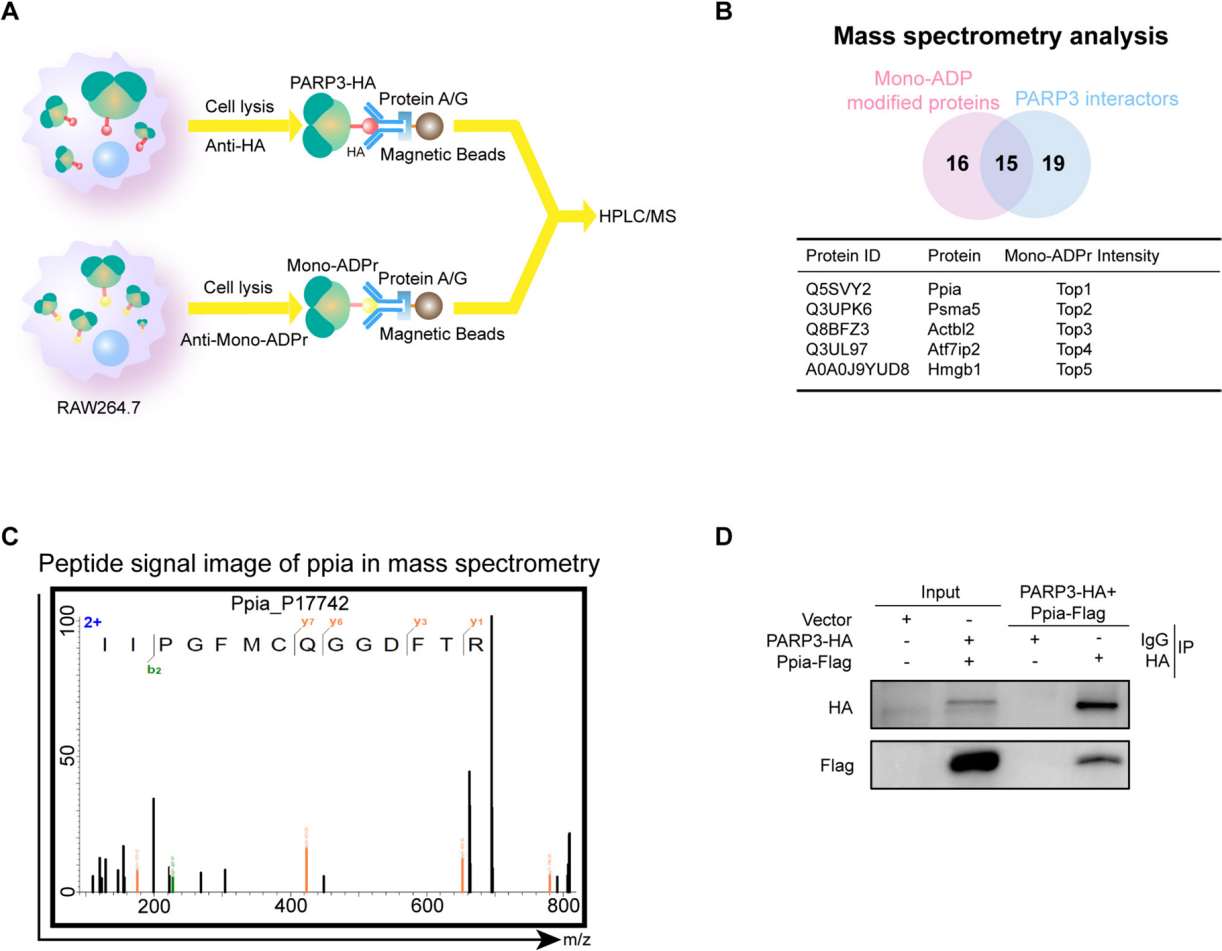
Ppia interacted with PARP3. **A** Schematic diagram of the screen for the candidate substrate of PARP3. **B** The top five proteins among the fifteen intersecting proteins were listed. These proteins were candidate substrates for PARP3. **C** The peptide map of the Ppia protein was presented. **D** Co-IP validated the interaction between PARP3 and Ppia

As PARP3 is a mono-ADP-ribosylation enzyme that promotes inflammation in macrophages, which relies on its catalytic activity, mono-ADP ribosylated substrate proteins must be involved in inflammation. Therefore, we screened the intersection of PARP3-interacting proteins and mono-ribosylated proteins in RAW264.7 cells. The physically interacting proteins of HA-tagged PARP3 (PARP3-HA) and mono-ribosylated proteins were pulled down via co-immunoprecipitation (Co-IP) and sequenced via mass spectrometry (Fig. 3A). Fifteen proteins were found to be mono-ribosylated and in contact with PARP3 (Fig. 3B). We then verified the top enriched protein, Ppia (CypA, Peptidyl-prolyl cis–trans isomerase A) (Fig. 3B-C), on the basis of its mono-ADPr intensity. PARP3 and Ppia interacted with each other in the Co-IP assay (Fig. 3D).

### Ppia mediated PARP3 to promote macrophage inflammation

**Fig. 4:**
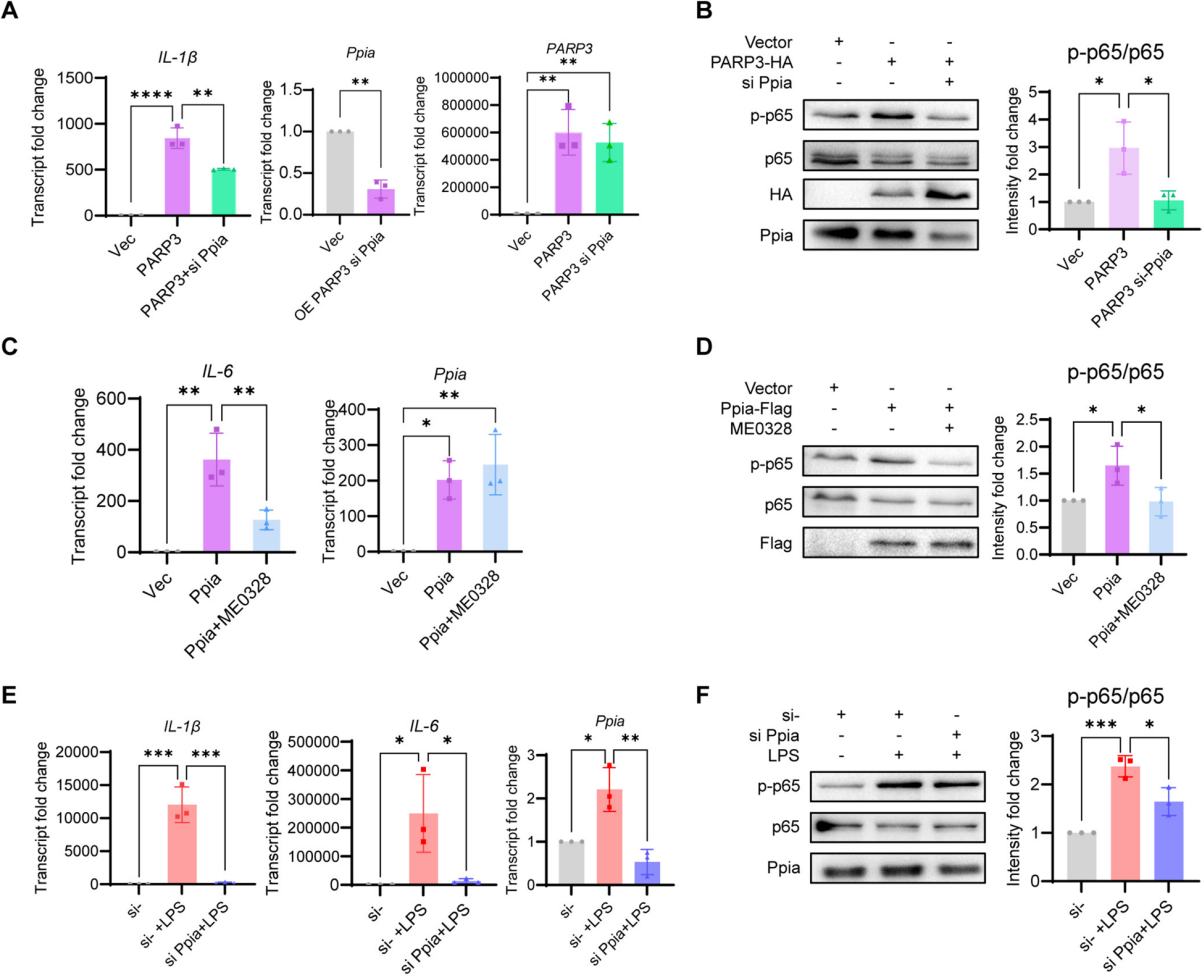
Ppia mediated PARP3 to promote inflammation. **A** The proinflammatory effect of PARP3 requires the mediation of Ppia. The expression of *IL-1β* was inhibited with the knockdown of *Ppia* (*n* = 3). **B** and the phosphorylation of p65 was also suppressed. (*n* = 3). **C** The presence of the PARP3 inhibitor ME0328 inhibited the increase in *IL-6* and **D** the phosphorylation of p65 induced by Ppia overexpression (*n* = 3). **E** LPS-induced *IL-1β* & *IL-6* expression (**F**) and p65 phosphorylation were inhibited by transfecting the siRNA of *Ppia* (*n* = 3). The mRNA levels of* IL-1β*, *IL-6*, *PARP3* and *Ppia* were detected by qPCR and protein levels of phosphorylated p65 were measured by western blot. The data were expressed as the means ± standard deviations. One-way ANOVA was used for statistical analysis in (**A**-**F**). *p < 0.05, **p < 0.01, ***p < 0.001 or ****p < 0.0001 were considered significant

To explore the relationship between PARP3 and Ppia, we simultaneously overexpressed PARP3 and knocked down *Ppia* in RAW264.7 and found its expression of *IL-1β* was significantly lower than that resulting from PARP3 overexpression alone (Fig. 4A). As Ppia was reported to activate inflammation via the NF-κB signaling pathway (Yang et al. 2022), p65 phosphorylation was shown to be induced by PARP3 overexpression, which was inhibited by the *Ppia* knockdown (Fig. 4B). Ppia overexpression induced *IL-6,* and treatment with ME0328 inhibited *IL-6* expression in a reverse manner (Fig. 4C). p65 phosphorylation was accordingly inhibited by ME0328 (Fig. 4D). *Ppia* knockdown also inhibited LPS-induced *IL-1β* & *IL-6* expression and p65 phosphorylation (Fig. 4E-F).

### Mono-ADP-ribosylation of Ppia at Glu140 promoted its activity and activate macrophage inflammation

**Fig. 5:**
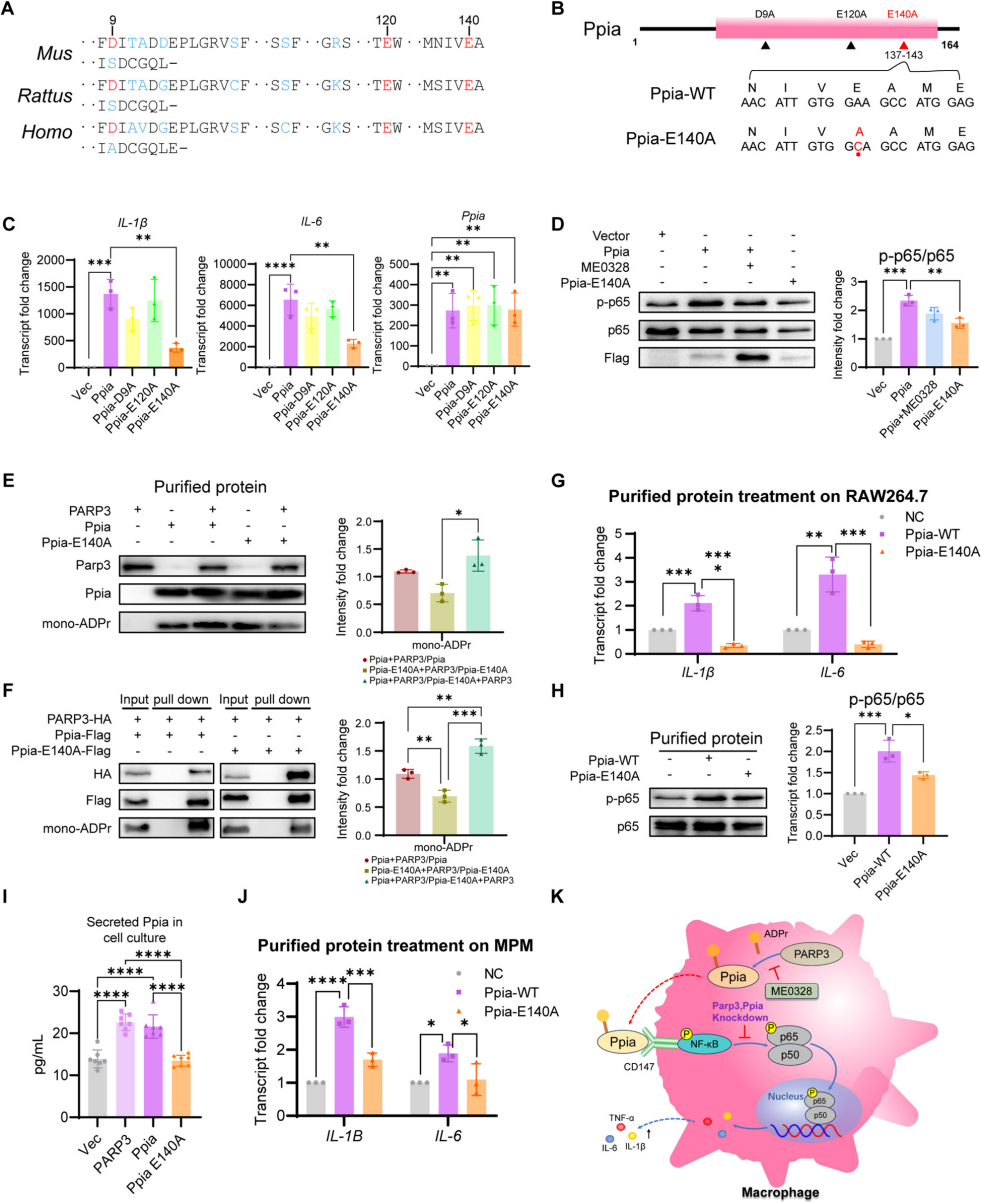
Ppia was mono-ADP ribosylated at the E140 residue by PARP3 to promote macrophage inflammation. **A** Schematic diagram of the homology of Ppia in mice, rats and humans. **B** Schematic diagram of the Ppia mutation sites. **C** Comparison of the effects of the D9A, E120A and E140A mutants on promoting inflammation (*n* = 3). The mRNA levels of* IL-1β*, *IL-6* and *Ppia* were detected by qPCR. **D** RAW264.7 cells were transfected with the Ppia-WT and Ppia-E140A plasmids, and their ability to stimulate inflammation was tested by determining the phosphorylation status of p65 (*n* = 3). Protein levels of phosphorylated p65 were measured by western blot. **E** The modification ability of the purified Ppia-WT and Ppia-E140A mutant proteins in the in vitro system was tested by western blot (*n* = 3). **F** Mono-ADP-ribosylation on Ppia E140 was confirmed by immunoprecipitation of Ppia and detection with a mono-ADPr antibody (*n* = 3). **G** The purified Ppia-WT and Ppia-E140A proteins were added to RAW264.7 cell cultures to detect the expression of *IL-1β* and *IL-6* by qPCR and (**H**) phosphorylation of p65 by western blot (*n* = 3). **I** The secretion of Ppia was influenced by mono-ADP ribosylation and was detected by ELISA (*n* = 7). **J** The purified Ppia-WT and Ppia-E140A proteins were added to MPM cell cultures to detect the expression of *IL-1β* and *IL-6* by qPCR (*n* = 3). **K** Schematic diagram of the mechanism by which PAPR3 mono-ADP ribosylate Ppia to stimulate the NF-κB pathway and activate inflammation in macrophages. The data were expressed as the means ± standard deviations. One-way ANOVA was used for statistical analysis in (**C-J**). *p < 0.05, **p < 0.01, ***p < 0.001 or ****p < 0.0001 were considered significant

Ppia is a substrate of PARP3 because it interacts with PARP3, responds to an inhibitor of PARP3, and mediates PARP3 to promote macrophage inflammation. On the basis of the prediction of the ADPr modification site (https://www.adpredict.net/), three homologous sites between humans, mice and rats were identified. The amino acids 9, 120, and 140 on Ppia were considered potential mono-ADP ribosylated residues. Mutations of Ppia-D9A, Ppia-E120A and Ppia-E140A were subsequently constructed (Fig. 5B). The mutants were transfected into RAW264.7 cells, which were subsequently compared with empty vector-transfected cells. The expression levels of *IL-6* and *Il-1β* were subsequently tested. We found that the E140A mutation disrupted the proinflammatory effect of Ppia (Fig. 5C). Ppia-E140A did not promote p65 phosphorylation, similar to what was observed in ME0328-treated RAW264.7 cells overexpressing Ppia (Fig. 5D). To confirm the mono-ADPr modification at the 140 residue of Ppia, which is catalyzed by PARP3, the His-tagged PARP3, Ppia-WT, and Ppia-E140A mutant proteins were purified from bacteria (PARP3) or from HEK293 T cells (Ppia-WT, Ppia-E140A). The in vitro catalytic system was constructed by mixing PARP3, Ppia, NAD^+^, and dsDNA to modify Ppia and test the modification by the mono-ADPr antibody. Quantitative analysis revealed enhanced mono-ADP ribosylation intensity in PARP3-modified wild-type Ppia compared to the E140A mutant (*p* < 0.05), as demonstrated by grayscale density measurements of immunoblot bands (Fig. 5E). Similar to the results of mono-ADP ribosylation of Ppia Assay in vitro (Fig. 5E), the quantitative analysis result of immunoprecipitation experiment in cell showed PARP3 mediated stronger mono-ADP ribosylation of wild-type Ppia than the E140A mutant, as evidenced by anti-mono-ADPr immunoblot densitometry (Fig. 5F). Because Ppia is secreted extracellularly and binds to CD147 on the cell surface to stimulate the NF-κB signaling pathway, the purified proteins of Ppia-WT or Ppia-E140A were added to RAW264.7 and MPM cell cultures to verify their ability to promote inflammation. Compared with Ppia-E140A, Ppia-WT induced more expression of *IL-1β* and *IL-6* at the mRNA level (Fig. 5G, J) and stronger p65 phosphorylation in RAW264.7 (Fig. 5H). The mouse CypA ELISA kit was used to detect the content of Ppia in the cell culture medium 48 h after transfection with PARP3, Ppia or Ppia-E140A overexpression plasmids. The result showed the secretion of Ppia in the group of Ppia-E140A was lower than that in the groups of PARP3 and Ppia, and similar to that of the empty vector transfected group (Fig. 5I).

### The Parp3 inhibitor ME0328 relieves ALI

**Fig. 6:**
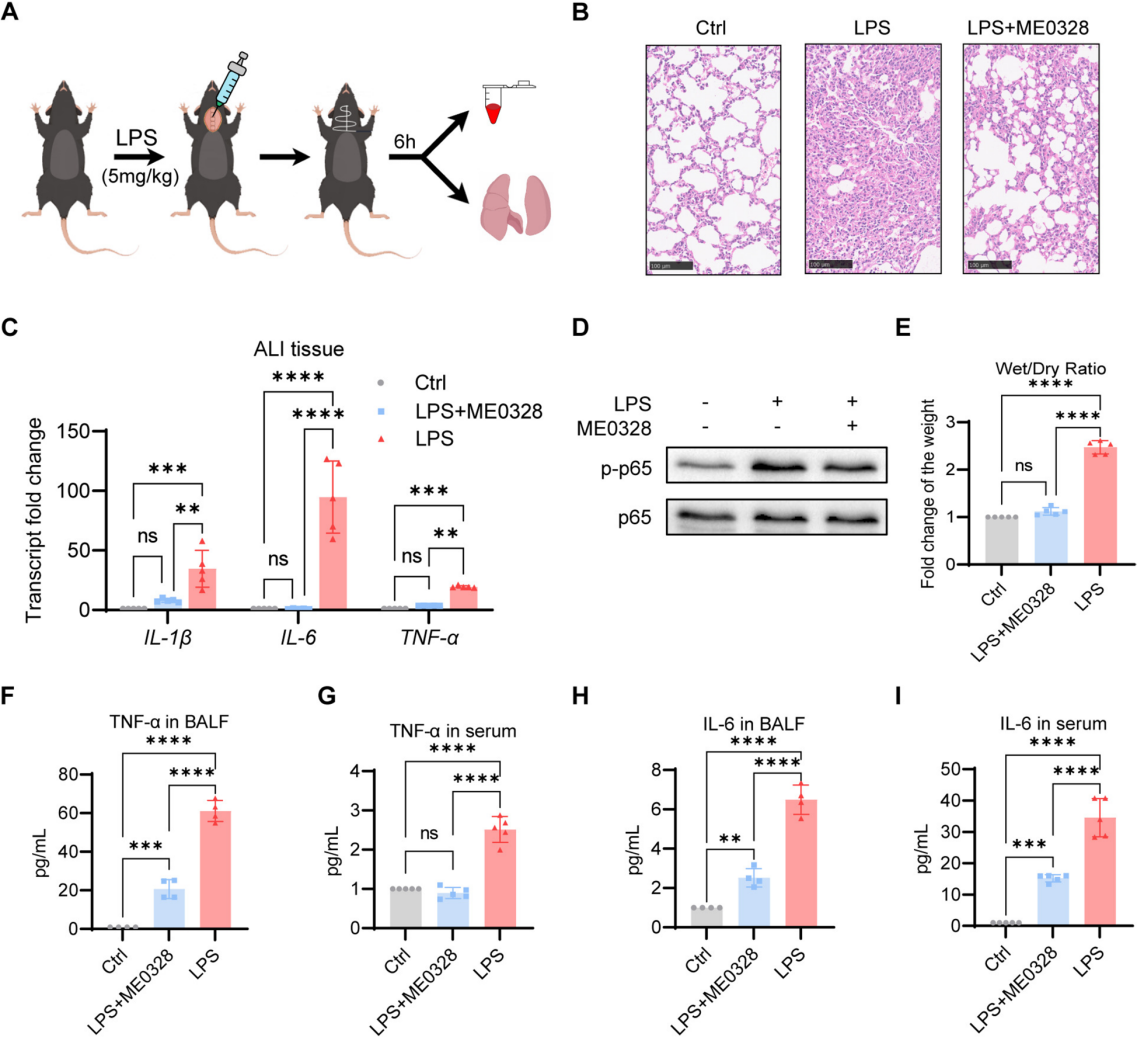
ME0328 inhibited ALI in mice. **A** Model construction and sampling diagram of acute lung injury in mice. **B** Hematoxylin‒eosin (H&E) staining of mouse lung tissues. **C** Expression levels of inflammatory factors (*n* = 5) detected by qPCR and (**D**) the phosphorylation of p65 in mouse lung tissues detected by western blot (*n* = 3). **E** Lung wet‒dry weight ratio (W/D) (*n* = 5). (**F**–**I**) The levels of the proinflammatory cytokines TNF-α and IL-6 in the BALF and serum were measured via ELISA (*n* = 5). The data were expressed as the means ± standard deviations. One-way ANOVA was used for statistical analysis in (**C**, **E**-**I**). *p < 0.05, **p < 0.01, ***p < 0.001 or ****p < 0.0001 were considered significant

We validated the effect of the Parp3 inhibitor ME0328 on LPS-induced acute lung injury in mice. Endotracheal administration of LPS has been widely accepted as a clinically relevant model of ALI (Ju et al. 2018) (Fig. 6A). Compared with the saline control, LPS significantly induced histological damage; however, ME0328 treatment protected the lung structure (Fig. 6B). The expression levels of inflammatory cytokines and p65 phosphorylation in the lung tissues of the mice in the ME0328-treated group were significantly inhibited (Fig. 6C, D). Furthermore, the lung wet‒dry weight ratio (W/D) was used to assess pulmonary microvascular permeability, an important feature of ALI (Wu et al. 2019). Compared with the LPS group, the wet‒dry weight ratio was significantly lower in the ME0328 group (Fig. 6E). The levels of the proinflammatory cytokines TNF-α and IL-6 in the serum and BALF were significantly inhibited by ME0328 (Fig. 6F to I).

## Discussion

In this study, Parp3 was upregulated in ALI tissue or RAW264.7 cell in response to stimulation with LPS. Parp3 is a mono-ADP-ribose transferase, and its catalytic activity contributes to the modification of Ppia. As mutation of the NLS of PARP3 significantly inhibits its proinflammatory function, the nuclear location of PAPR3 should be important for the stimulation of PARP3 activity. Damaged DNA is a stimulator of PAPR3, so the DNA damage triggered by inflammation should be required for suitable PAPR3 location and activity.

Ppia is a known secreted proinflammatory factor and is considered a marker for rheumatoid arthritis (RA), sepsis, asthma, and periodontitis. Ppia is highly expressed in LPS-activated macrophages and stimulates the NF-κB signaling pathway via the cell membrane receptor CD147 in the activation phase of acute inflammation. Here, we demonstrated that Ppia is a substrate of PARP3. Ppia affects PARP3 to promote the transformation of macrophages to the M1 pro-inflammatory phenotype, activating the NF-κB signaling pathway, and releasing a large amount of pro-inflammatory cytokines (IL-1β, IL-6). PARP3 catalyzes the mono-ADP-ribosylation of Ppia and stimulates the NF-κB signaling pathway to promote an inflammatory storm that leads to acute lung injury. Mono-ADP-ribosylation usually affects the function of substrate proteins by adding negatively charged ADP-ribose to change the structure of the substrates (Schreiber et al. 2006; Szabo et al. 2020). This is different from poly-ADP-ribosylation, which provides a"platform"to recruit partners via poly-ribosylation branches. The structural alteration of Ppia by mono-ADP-ribosylation might affect its secretion or contact with CD147 (Kosugi et al. 2015). The functional alteration of mono-ADP-ribosylated Ppia is worth further study. There is limited clarity on where Ppia is modified within the cell, and we suggest two possibilities: 1. PAPR3 is activated by damaged DNA in the nucleus and is transferred to the cytoplasm to modify Ppia. 2. Ppia is modified in the nucleus. The locations of PAPR3 and Ppia in addition to the LPS treatments should be studied further.

To date, the relationship between PARP3, Ppia and the NF-κB pathway has not been fully clarified. In our study, we detect that PARP3 is the upstream regulator of Ppia and identify the mono-ADP-ribosylation site of Ppia induced by PARP3. Then we study the functional significance of this mono-ADP-ribosylation in detail. It appears that Ppia mono-ADP-ribosylation at E140 significantly enhances the transcription activity of NF-κB toward multiple target genes and promoting ALI development. On the other hand, we demonstrate that Ppia is mono-ADP-ribosylated by PARP3 and the MARylation of Ppia significantly activates its secretion and inflammation function in macrophage inflammation. Our findings may provide insights into the mechanism by which macrophages play a pathogenic role in the progression of ALI. Constructing a genetically engineered mouse with ribosylation mutations on Ppia-E140 would be valuable for detecting the impact of post-translational modifications of Ppia on ALI.

PTM is a vital process after the translation of proteins, usually dictates the activity or function of the target proteins (Hong et al. 2018). Although we demonstrated that PARP3 directly mono-ADP-ribosylated Ppia, it is not entirely understood how this MARylation enhances the secretion of Ppia. Ppia has been shown to be binding the Ig2 domain of CD147 extracellularly and altering the inherent cis:trans equilibrium of CD147 at Pro211 (Schlegel et al. 2009). This maybe one of the ways to promote ALI through the NF-κB signaling pathway.

The E140 site of Ppia was modified in our study. However, three sites were predicted, only the E140A mutation strongly affected its proinflammatory effect, but the other two mutants weakly affected the function of Ppia. The mutation at E140 did not abolish its proinflammatory function. Ppia E140A still promotes cytokine expression. These findings suggest that all three mono-ADP-ribosylation sites on Ppia regulate its function and that the E140 residue is the dominant functional residue. Although our data demonstrated that E140 is major site of mono-ADP-ribosylation mediated by PARP3, the MARylation of Ppia is not completely eliminated. This suggests that the other two mono-ADP ribosylation sites may have functions other than inflammation.

Given that PARP3 inhibitor is currently used to treat cancer (Sharif-Askari et al. 2018). Our observation that ME0328 suppressed ALI might be an important translational consequence. Parp3 is overexpressed in ALI and macrophage inflammation. In summary, our results, together with previous work from others, reveal a different function of the Ppia/PARP3/NF-κB feedback in ALI. The ability of the PARP3 inhibitor to suppress ALI suggests the potential application of PARP3 as a therapeutic target for ALI.

## Conclusions

In summary, we identified five elevated PARPs in the lung tissues of mice with acute lung disease, all of which catalyze mono-ADP ribosylation. The expression of PARP3 was significantly elevated, and no literature has reported that PARP3 is associated with inflammation. The expression and pro-inflammatory effect of Parp3 were enhanced by macrophage in ALI lung tissue. An upregulation of Parp3 was detected in macrophages under ALI conditions, contrasting with its low constitutive expression in control macrophages. This indicates that Parp3 in macrophages may have an effect on the development of ALI. Parp3 inhibitor ME0328 alleviated the inflammatory response and histopathological damage in macrophages and ALI lung tissues. Therefore, Parp3 in macrophages could be a therapeutic target. We demonstrate that Parp3 interacts with Ppia and modifies it to influence inflammatory response and Ppia secretion function. In addition, our results show that Parp3 requires activation of the precursors to have the ability to catalyze modification. To verify whether Parp3 interacts with Ppia in the nucleus or cytoplasm, more independent studies and multiple methods are needed. Overall, these findings imply that the PARP3-Ppia-NF-κB axis might be a promising therapeutic target in degenerative retinal diseases.

## Data Availability

The datasets supporting the conclusions of this article are available in the NCBI repository, unique persistent identifier and hyperlink to datasets in https://www.ncbi.nlm.nih.gov/bioproject/?term=PRJNA1224952.

## Abbreviations

ALI: Acute lung injury
ARTD: ADP-ribosyltransferase diphtheria toxin-like
BALF: Bronchoalveolar lavage fluid
CAT: Catalytic activity domain
CD147(BSG): Basigin
Co-IP: Co-Immunoprecipitation
CXCL10: C-X-C motif chemokine ligand 10
DEGs: Differentially expressed genes
DMEM: Dulbecco’s Modified Eagle Medium
ELISA: Enzyme-linked immune sorbent assay
FBS: Fetal bovine serum
GO: Gene ontology
H&E: Hematoxylin-eosin
HRP: Horseradish Peroxidase
IL-1β: Interleukin-1β
IL-6: Interleukin-6
KLF7: Krueppel-like factor 7
Krt7: Keratin 7
LPS: Lipopolysaccharide
MART: Mono-ADP‒ribose transferase
MIP-2: Macrophage inflammatory protein-2
MPM: Mouse primary peritoneal macrophage
MVs: Micro-vesicles
NAT: N-acetyltransferase
NF-κB: Nuclear factor-κB
NLS: Nuclear localization sequence
PARP: Poly ADP-ribose polymerase
PBS: Phosphate buffered saline
PCR: Polymerase Chain Reaction
PFA: Paraformaldehyde
PMSF: Phenyl methane sulfonyl fluoride
Ppia: Peptidyl-prolyl cis–trans isomerase A
PTMs: Posttranslational Modifications
PVDF: Poly-vinylidene fluoride
ROS: Reactive oxygen species
RT-qPCR: Real-time quantity PCR
TFs: Transcription factors
TFRC: Transferrin receptor
TLR: Toll-like receptor
TMB: 3,3’,5,5’-Tetramethylbenzidine
TNF-α: Tumor necrosis factor alpha
WB: Western Blot
